# Intradural Extramedullary Thoracic Cavernoma in a Man with Familial Multiple Cavernomas

**DOI:** 10.7759/cureus.3115

**Published:** 2018-08-07

**Authors:** Robert Ziechmann, Stephanie Zyck, Satish Krishnamurthy, Michael Galgano

**Affiliations:** 1 Neurosurgery, Temple University, Philadelphia, USA; 2 Neurosurgery, State University of New York Upstate Medical University, Syracuse, USA

**Keywords:** intradural extramedullary cavernoma, familial multiple cavernoma syndrome, thoracic radiculopathy, cavernoma

## Abstract

Intradural extramedullary cavernomas are rare vascular malformations of the spine. Of the 40 previously described cases, four involved the upper thoracic spine and one was associated with familial multiple cavernoma syndrome. We report the case of a 55-year-old man with familial multiple cavernomas presenting with thoracic radiculopathy and back pain due to a T3-T4 intradural extramedullary cavernoma compressing the spinal cord and dorsal nerve rootlets. Vascular malformations of the thoracic spine are a rare cause of atypical chest pain that should be considered in an individual with familial multiple cavernoma syndrome.

## Introduction

Cavernomas are uncommon vascular malformations that may be found throughout the central nervous system. Spinal cavernomas are less common, as they make up only 5% of spinal vascular malformations [1]. Intradural extramedullary cavernomas are rarer still. Our review of the literature yielded a total of 40 previously described cases. Four of these cases involved the upper thoracic spine, and all had in common a presentation involving subarachnoid hemorrhage [2-5]. We describe a case without a subarachnoid hemorrhage. In addition, this a case of intradural extramedullary cavernoma in the context of familial multiple cavernous malformation syndrome.

## Case presentation

**Figure 1:**
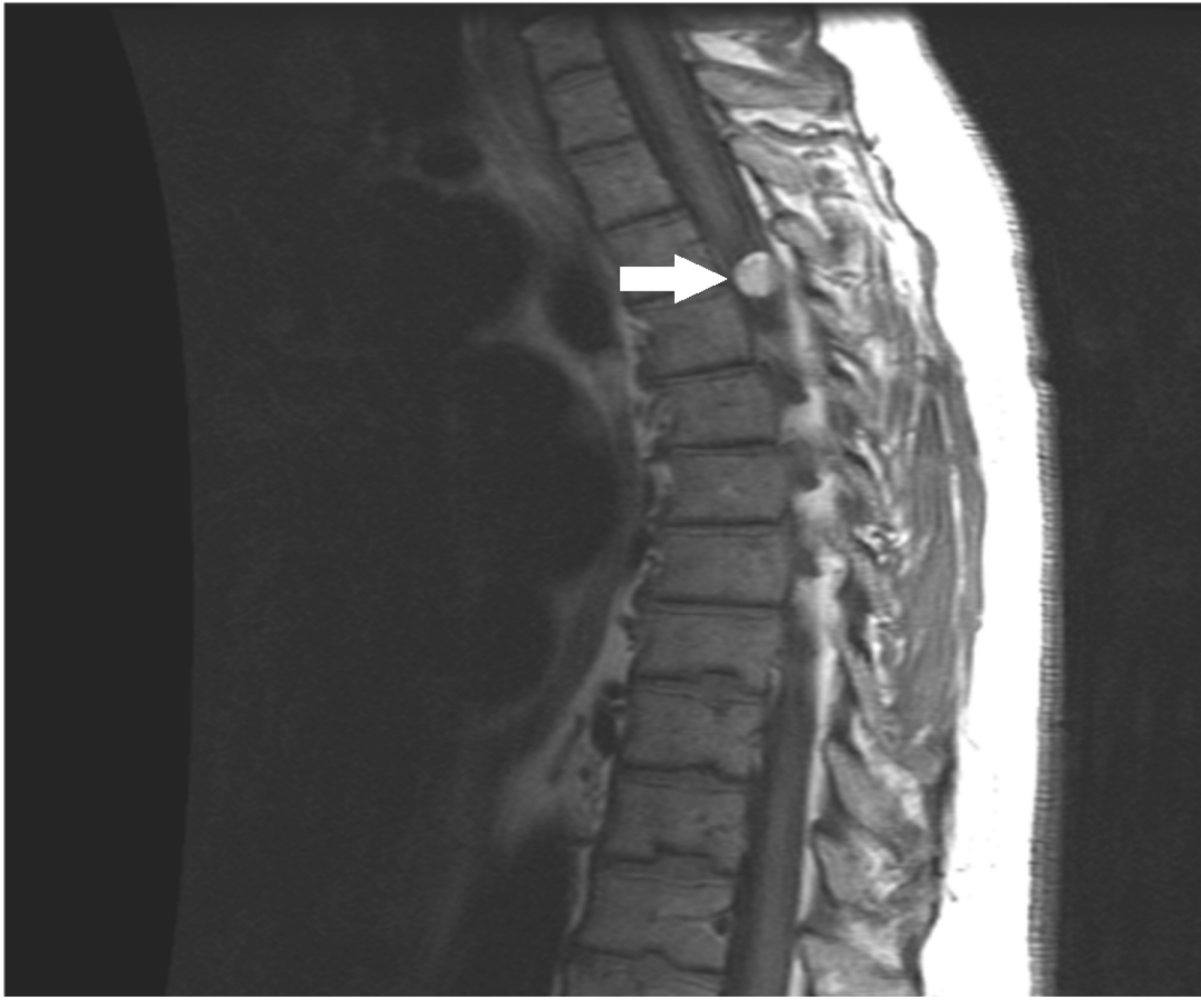
Preoperative T1 sagittal MRI showing a hyperintense lesion at the T3-T4 level MRI: magnetic resonance imaging

**Figure 2:**
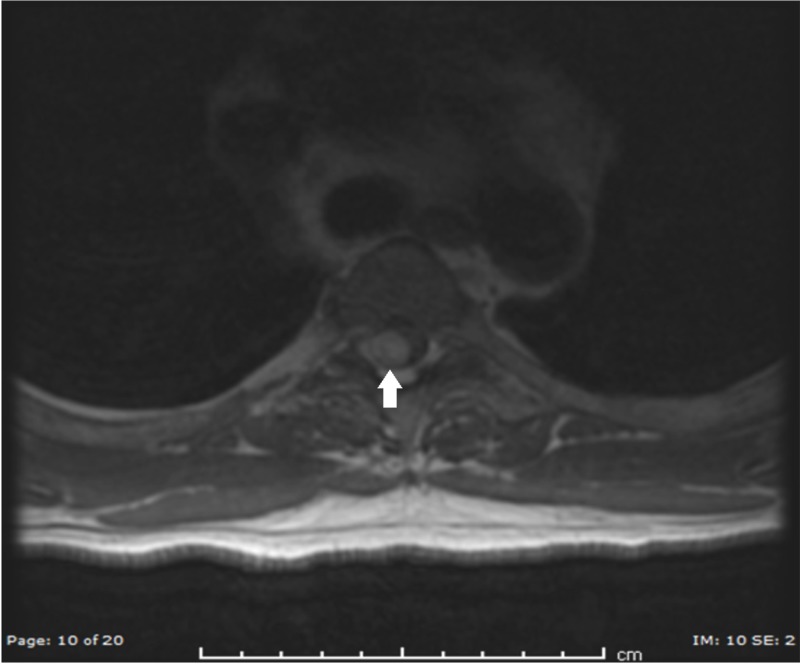
Preoperative T2 axial MRI showing a hyperintense lesion at the T3-T4 level MRI: magnetic resonance imaging

A 55-year-old male with a history of multiple cerebral cavernomas presented to the emergency department of an outside hospital for back pain radiating to the right chest region. He attributed his pain to muscle strain associated with fixing a popup camper. An extensive workup was done and was negative except for magnetic resonance imaging (MRI) of the spine. This showed evidence of an intradural extramedullary lesion at the T3-T4 level, located dorsally and directed rightward. The lesion appeared hyperintense on T1 and T2 with compression of the cord (Figure 1 and Figure 2).

The patient was referred to the neurosurgery clinic, where he had been seen one month prior for decreasing dexterity of the left hand of one year’s duration. The past medical history was significant for seizures beginning at age 15, for which the patient underwent separate partial resections of the right frontal and temporal lobes. Multiple new cavernomas had been found following a breakthrough seizure at age 50. When the patient first presented to the neurosurgery clinic at age 54, he reported a decrease in left-hand dexterity. The only interval change in the MRI at the time was an enlargement of a right porencephalic cyst in the context of the patient’s previous surgeries.

Neurological exam revealed right-sided hyperreflexia but no weakness of the upper or lower extremities. He was diagnosed with thoracic myelopathy. Given the symptomatic presentation with severe radiculopathy and cord compression, the patient was offered surgery.

**Figure 3:**
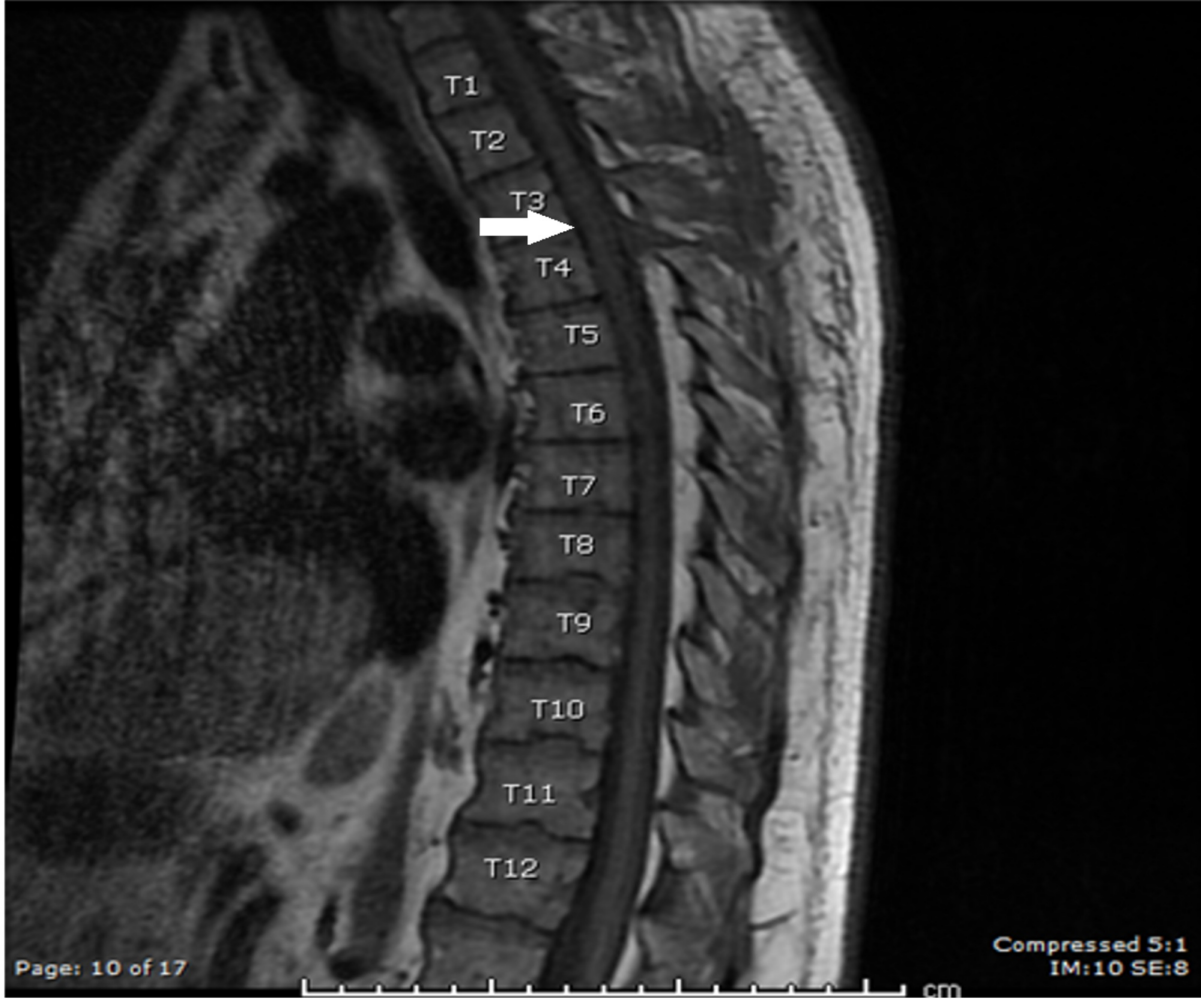
Postoperative T1 sagittal MRI with contrast MRI: magnetic resonance imaging

Under general anesthesia with neuromonitoring, a T3-T4 hinge laminotomy was performed. Ultrasound was used to confirm the location of the lesion within the dura. Under the magnification of the operating microscope, a curvilinear durotomy was performed. The lesion was hemorrhagic and highly friable. It appeared to be attached to the T3 dorsal nerve rootlets. Gross total resection was achieved in a piecemeal fashion using tumor forceps. After the tumor was mobilized off the spinal cord and the inner surface of the dura, it was seen to have left an impression on the thoracic spinal cord in the T3-T4 area. The durotomy was then closed in a water-tight fashion and the wound was closed in multiple layers. No complications were noted during the procedure and no changes were seen on neuromonitoring. Postoperative scans demonstrated no residual lesion (Figure 3). The patient was relieved of his mid-back or thoracic radiculopathy and remained neurologically at baseline immediately post-operatively and at six months.

## Discussion

Spinal intradural extramedullary cavernomas are a rare entity with only 40 cases reported in the literature [2,6-7]. Age at presentation ranged greatly from 20 to 79 (mean 49.7). The clinical manifestations of spinal cavernomas varied nearly as much as patient age and lesion location. Sensory dysfunction was the most common at 40%, followed by motor dysfunction at 35% and back pain at 35%. Subarachnoid hemorrhage, sciatic pain, autonomic dysfunction, hydrocephalus, dizziness, and cognitive dysfunction were also reported.

In the vast majority of cases, neurologic impairment was due to a localized mass effect. In cases where the origin of the cavernoma was described, the majority had an origin in the spinal nerve root [8-9]. Onset is generally subacute with progression over time. Not all cases involved a local effect, however. Four patients presented with isolated subarachnoid hemorrhage [3-4,8]. This is of particular interest to our present case because all previous intradural extramedullary cavernomas of the upper thoracic spine involved a component of subarachnoid hemorrhage [2-5]. Other indirect pathophysiology involved intra-canal hemosiderosis, which potentially led to hearing loss, dizziness, and hydrocephalus.

A provisional diagnosis of intradural extramedullary cavernoma may be made with a spinal MRI. Imaging can localize the lesion by detecting blood as well as breakdown products, such as hemosiderin, although as noted in one paper, hemosiderin is less abundant in epidural and in intradural extramedullary cavernomas compared to intramedullary cavernomas [10]. A definitive diagnosis can be made on histopathology, showing a mass of irregularly dilated blood vessels with thin walls.

While the etiology, pathophysiology, and clinical presentation of intradural extramedullary spinal cavernomas may vary, it is clear that the outcome following surgical resection (the gold standard of treatment) is overwhelmingly favorable. Total resection was achieved in the vast majority of cases [9]. The neurological outcome was described as excellent for all but five patients [6,9].

While the prognosis of intradural extramedullary cavernomas may be quite favorable, it is worth considering the possibility of other cavernomas elsewhere along the neuraxis in a patient with a previous cavernoma or a family history of cavernoma. The presence of cerebral cavernomas may add significant morbidity to the overall prognosis. For a patient with a family history of cavernomas, further imaging, chromosomal testing, and genetic counseling may be warranted based on clinical judgment.

## Conclusions

Thoracic radiculopathy is an unusual cause of atypical chest pain. In addition to what is typically described as a band-like chest pain, a mechanical compression of the thoracic nerve root may also cause motor weakness and sensory disturbances. In a patient with atypical chest pain and syndromic vascular malformation, such as familial multiple cavernoma syndrome, a structural cause of atypical chest pain should be considered in the differential diagnosis.
